# *In-Situ* LED-Based Observation of Snow Surface and Depth Transects

**DOI:** 10.3390/s20082292

**Published:** 2020-04-17

**Authors:** Celeste Barnes, Chris Hopkinson, Thomas Porter, Zhouxin Xi

**Affiliations:** Department of Geography, University of Lethbridge, Lethbridge, AB T1K 3M4, Canada; celeste.barnes@uleth.ca (C.B.); portta@uleth.ca (T.P.); zhouxin.xi@uleth.ca (Z.X.)

**Keywords:** Leddar, snowpack depth, LIDAR, LED LIDAR, sonic ranging device, intensity

## Abstract

As part of a new snowpack monitoring framework, this study evaluated the feasibility of using an LED LIDAR (Leddar) time of flight sensor for snowpack depth measurement. The Leddar sensor has two additional features over simple sonic ranging sensors: (i) the return signal is divided into 16 segments across a 48° field of view, each recording individual distance-to-target (DTT) measurements; (ii) an index of reflectance or intensity signal is recorded for each segment. These two features provide information describing snowpack morphology and surface condition. The accuracy of Leddar sensor DTT measurements for snow depth monitoring was found to be < 20 mm, which was better than the 50 mm quoted by the manufacturer, and the precision was < 5 mm. Leddar and independent sonic ranger snow depth measurement showed strong linear agreement (*r*^2^ = 0.98). There was also a strong linear relationship (*r*^2^ = 0.98) between Leddar and manual field snow depth measurements. The intensity signal response was found to correlate with snow surface albedo and inversely with air temperature (*r* = 0.77 and −0.77, respectively).

## 1. Introduction

Wintertime snow accumulation and associated snowmelt provide a significant contribution to water resources in regions with seasonal snow packs [1] as surface water is “locked up” in a frozen storage state [2,3]. Snow depth and density measurements are required to quantify the amount of snow water equivalent (SWE) that will be released from the snowpack at the time of melt. Of the two variables, snow depth is the major component of SWE [4]. Alberta Rocky Mountain headwater snowpack monitoring [5] has been operational for several decades, using both destructive and non-destructive techniques [6]. A snow probe and weighing tube are used to obtain field validation depth and SWE [7,8]. This provides an accurate single point measurement but disturbs the snowpack in the process, making future measurements difficult to repeat at the same location. The second operational monitoring method in the headwaters uses non-destructive sonic ranging devices [9] to measure snowpack depth [10,11]. Such units can be mounted on a tower pointing over the ground surface. Ultrasonic pulses are emitted and echoes are received by the sensor. The return signal is used to calculate snow depth by differencing distance-to-target (DTT) measurements when the snow surface is present from the DTT observation of the no-snow ground surface, following air temperature compensation. These sensors have a high degree of accuracy, but depth is an average obtained from the total sensor footprint (the further from the target, the larger the footprint) including undulations in the ground and snow surface. Snowpack depth is continuously recorded over the snow season. Sonic ranging sensors do not describe the snowpack morphology, structure, or density characteristics. 

The intent of this study was to examine a low-cost (≈1000 Canadian dollars) and low-power (4 watt) alternative to the contemporary sonic ranging snow depth sensor. The low-cost low-power Leddar Tech IS16 (hereafter referred to as Leddar) sensor is a LIDAR (light detection and ranging) based “time of flight” device. It emits a single LED (light emitting diode)-diffused light source beam in the near infra-red (NIR) 940 nm wavelength. The return signal is divided into 16 segments. Both temperature compensated DTT and light intensity are observed and measured for each segment. The Leddar unit has added features over the sonic ranging sensor, those being a DTT and intensity response for each of the 16 segments of its footprint. There is limited light transmission into the surface of natural snowpacks [12], making LED technology an ideal candidate for snow depth monitoring by calculating the difference of the DTT snow surface from the bare earth DTT. Sonic ranging sensor pulses penetrate freshly fallen snow, producing an underestimation of snowpack depth [11,13]. Beyond evaluating the potential to observe snow surface height and snowpack depth, a further goal of this study is to evaluate which, if any, snowpack features can be inferred from the additional intensity signal attribute collected by LED ranging sensors.

The snowpack surface texture and reflectance change over time as the pack evolves due to increasing and decreasing depths from snow accumulation, ablation, compaction, and wind/gravity-induced redistribution [14,15]. As the snowpack evolves, variation in crystal grain size and structure [16,17,18], density, and the amount of water within the pack occurs [19,20]. Ice lenses form within the pack during melt and refreeze cycles [6]. Impurities such as dust or dirt settle in the snowpack [19]. Coalesced and/or wet snow has a lower albedo than freshly accumulated snow [21]. As the snowpack ages and eventually becomes isothermal, the reflectance signature changes [22,23]. 

The spectral response or albedo of snow is dependent on snow crystal grain size, age of the snow, and amount of water and impurities in the pack [12]. This causes the spectral response to be different at various stages of metamorphism. “Fresh” new snow produces the highest spectral reflectance over aging snow, soils, and vegetation [16] in the 940 nm band, in which the Leddar unit operates. At the 940 nm band, spectral albedo ranges from approximately 0.5 to 0.9. The continuous spatiotemporal intensity signal has the potential to be used to detect changes in snowpack surface characteristics. For example, it should be possible to use the intensity signature to detect when snow is falling on the pack’s surface, since fresh snow has a higher reflectance than older snow. These variations in the snowpack should be detectable in the Leddar’s DDT and intensity signals. The expectation is to see an increase in the spectral response for new snow accumulation and a decrease as the snowpack compacts and metamorphoses over time. 

This study assessed the performance and features of the Leddar sensor. The objectives were to: (1) examine the Leddar sensor performance and the controls on the Leddar intensity signal; (2) compare the continuous temporal snowpack depth measurements of the Leddar with a standard sonic ranging sensor and infrequent field depth data observations to quantify sensor precision and accuracy for this particular application. 

## 2. Materials and Methods

### 2.1. Leddar and SR50A Sensor Specifications

**Figure 1:**
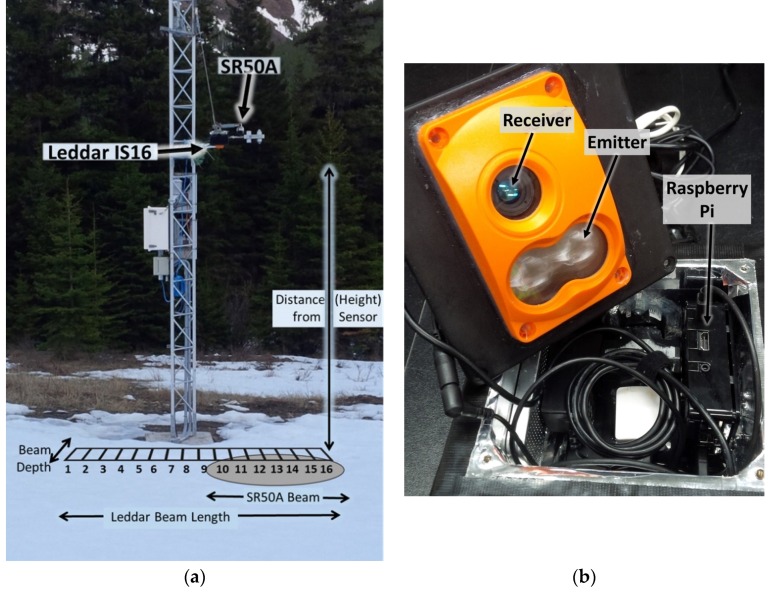
(**a**) Leddar and SR50A sonic ranging device co-located on a tower. Approximation of sensor beam footprint; not to scale. Leddar segment 1 orientation is the north side of the tower. The sensor emits defuse LED light and receives return signals for 16 segments. The ground surface footprint is dependant on the height of the sensor above the target, and the beam length and depth. (**b**) LeddarTech IS16 pulsed based time of flight LED LIDAR sensor logging to a Raspberry Pi 3.

The Leddar and SR50A sonic ranging sensors used in this study each capture DTT measurements. An approximation of the respective Leddar and SR50A DTT sampling areas is shown in Figure 1a. The LeddarTech IS16 sensor (Figure 1b) is a solid-state, pulse-based, time-of-flight LED LIDAR ranging instrument that receives return signals on a 16-channel photodetector [24]. The unit’s internal processing chip performs a full waveform analysis on all segments of the return signal. When the “object demerging” feature is enabled, it is possible for the sensor to detect multiple objects at varying distances and intensities within its field of view (FOV). LED technology is sensitive to ambient temperature [25], and an inverse relationship exists between external temperature and light output (illumination) [26]. A temperature sensor is located on the Leddar circuit board near the emitter optics [24,27]. Proprietary algorithms compensate for this temperature sensitivity and are used to calculate distance measurements within the manufacturer’s specified precision and accuracy [28].

The photodetector chip on the Leddar sensor has limited distance detection [27] and uses the intensity (amount of light captured by the receiver) property as part of the calculation for detecting multiple objects at varying distances within the FOV. The Leddar sensor range is zero to 50 m (Table 1). The sensor has a 48° × 8° sampling FOV. The ground surface footprint (seen in Figure 1a) is dependant on the height of the sensor above the target and defined by the beam length and depth. The length of the beam on the ground is 0.89 multiplied by the distance to the target. Each segment is 1/16 of the total beam length. The depth of the beam is 0.14 multiplied by the distance of the sensor to the target. 

LeddarTech technology is used for industrial applications, such as vehicle collision detection, in which fast response times from the sensor are required [27]. The distance measurement accuracy reported by the manufacturer is 50 mm, the precision of the sensor (if the intensity return signal is greater than the manufacturer’s specification of 15) is 6 mm, and the resolution is 10 mm. For this study, the Leddar sensor was statically mounted over the ground surface at a fixed height of 2.93 m with a calculated beam length of 2.61 m (each segment length is 0.16 m) and depth of 0.41 m. In comparison, the SR50A sensor emits ultrasonic pulses. The sensor’s footprint on the ground surface is circular with an approximate radius of 0.27 multiplied by the height of the sensor above the target surface. A temperature corrected measurement for DTT is averaged over the entire footprint as a single reading. The distance range of the SR50A is 0.5 to 10 m. The SR50A DTT measurement accuracy as reported by the manufacturer is 12 mm on a 3 m high mount, and the resolution is 0.25 mm. A comparison of the technical specifications of Leddar and SR50A units is shown in Table 1. 

### 2.2. Field Deployment Setup, Configuration, and Data

**Figure 2:**
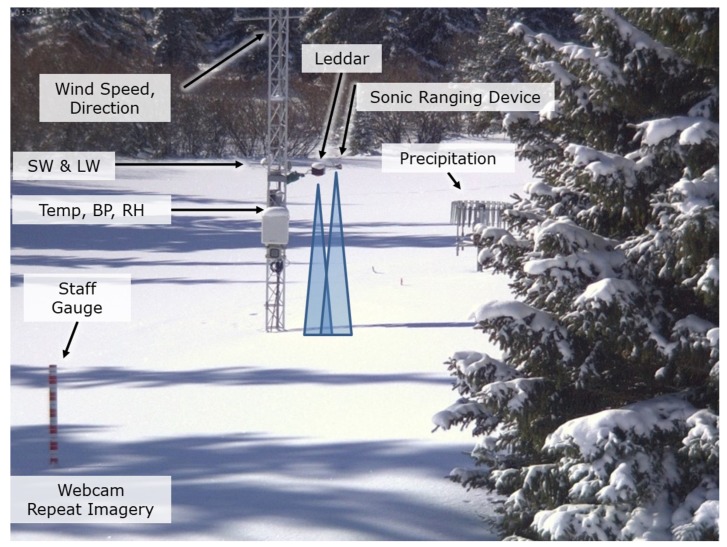
The West Castle Field Station tower and snow depth monitoring sensors (Leddar and SR50A), the totalizing precipitation gauge located south of the tower, and the meteorological sensors for the 2017–2018 snow season collecting wind speed, wind direction, air temperature (temp), barometric pressure (BP), relative humidity (RH), and incoming and reflected shortwave (SW) and longwave (LW) radiation. A temperature sensor was located at the ground surface to collect ground temperature.

Leddar and SR50A instrument testing and snowpack validation were completed at the University of Lethbridge West Castle Field Station (WFS), located in the headwaters of the Oldman River Basin, Alberta, Canada from 2017 (December 14) to 2018 (April 27). The Leddar unit was mounted on the weather station tower coincident with a SR50A such that the beams of the two sensors partially overlapped, as shown in Figure 1a. The ground surface beneath the Leddar and SR50A sensors had a downward slope of 0.07 m over a distance of 3.0 m. Meteorological data was collected at the tower site for wind speed, wind direction, temperature (temp), barometric pressure (BP), relative humidity (RH), and incoming and reflected shortwave (SW) and longwave (LW) radiation (Figure 2). A temperature sensor under the Leddar unit at the ground surface collected ground temperature. A totalizing precipitation gauge located nine meters south of the tower provided cumulative precipitation data. 

To validate Leddar and SR50A sensor snow depth measurements, eleven site visits took place from December 21 to April 27. Biweekly to monthly field measurements occurred during the months of December to March. At the onset of snowmelt, sampling was done at a weekly to daily time interval. Snow depth field measurements using a graduated avalanche probe were taken by standing behind the tower out of the sensor field of view and sampled directly under the Leddar unit. Field depth measurements collocated and coincident in time with the Leddar deployment were collected to support and validate both the Leddar and SR50A measurements. Leddar, SR50A, and meteorological data were extracted from the 15-minute timestep dataset for a four-hour time period when field measurements occurred. The mean, minimum, and maximum were calculated for the SR50A. The mean for the Leddar was derived from the mean value of all segments. Minimum and maximum were extracted based on the range of values from all segments of the Leddar sensor.

#### 2.2.1. LeddarTech IS16 (Leddar) Configuration and Laboratory Calibration

The Leddar configuration settings determine the accuracy, precision, and resolution of the emitted and return signals for DTT and intensity measurements (Table 2). The Leddar’s LED pulse rate is 102.4 kHz. The parameter configuration selected maximized the accuracy and precision of object detections that resulted in a maximum range of 21.3 m [24]. “Crosstalk removal” and “object demerging” were both enabled to reduce return signal degradation from objects detected in other segments.

**Figure 3:**
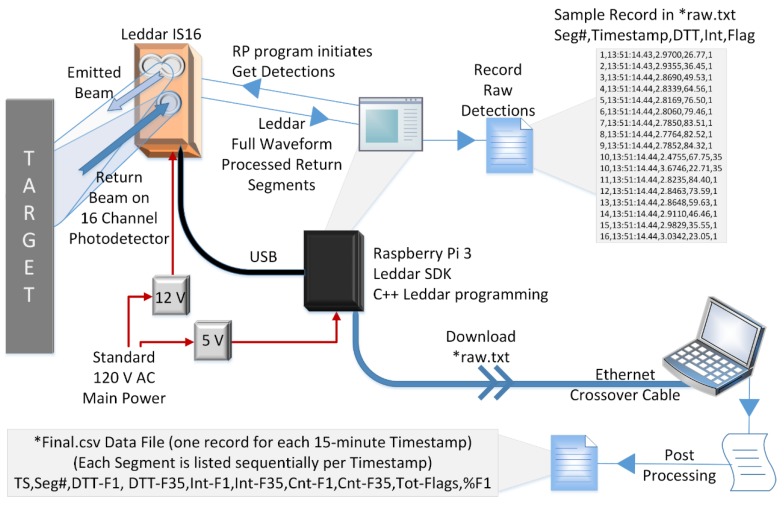
Data flow diagram of the Leddar-Raspberry Pi for the 2017−2018 Snow Season. TS refers to timestamp, Seg# refers to segment number, DTT-F# is the distance to target for flag number, Int-F# is the intensity for flag number, Cnt-F# is the number of returns received for the status flag number, Tot-Flags is the total count of both flag numbers, and %F1 is the percentage of Flag = 1 returns for the segment.

The control and logger system for the Leddar sensor had a similar design to that of [30], wherein the Leddar unit was used for 3D digital canopy foliage sampling. The Leddar unit (Figure 1b) was connected to a Raspberry Pi 3 (RP) running the Raspbian OS through a 2.0, 12 Mbits/s USB cable (Figure 3). A computer program [31] executed Leddar SDK (software development kit) commands to obtain DTT, intensity, and status flag for a 15-minute time step. At the start of the collection interval, the Leddar unit was “turned on” to acquire continuous pulse measurements for a period of one minute. For each measurement, the 16-channel photodetector divided the return signal into separate segments where a full waveform analysis was performed by the Leddar onboard processing chip. The resulting measurement was returned to the RP. The program then went into a “wait” state for the next 14 minutes. 

The RP program converted Leddar range measurements (R) into vertical distance (V_T_) [32] values using Equation (1).

V_T_ = (R_i_ + R_c_) cos(φ) cos(α + β(i − 8.5) + γ(i − 8.5)^2^) (1)

R_i_ is the range measurement of the i^th^ segment, R_c_ is the fixed shift of the range measurement from the true range, φ is the fixed zenith boresight shift, α is the azimuth boresight shift, β is the i^th^ segment’s angle deviation from the boresight on Leddar beam plane, and γ is the 2^nd^ order non-linear angle deviation (8.5 is the offset to the center of segments 1 to 16). Leddar calibration parameters were determined in the laboratory. The Leddar unit was mounted facing a flat level reference surface with no zenith deviation (φ = 0). Measurements were taken at varying boresight angles (α) and vertical distances (V_T_) from Leddar optical emitter center to the reference surface. R_c_, α, β, and γ are the calibration parameters. V_T_, R_i_, and φ are known values. This made it possible to infer R_c_, β, and γ from Equation (1) based on the Gauss–Newton algorithm with Huber robust function [33] to get the best parameter fit in order to evaluate the accuracy of the Leddar measurement. 

Two data files were created: the first containing the raw measurements per pulse for each of the 16 segments stored on the RP (see Figure 3 *raw.txt Data File at the top right of the diagram); the second created from the downloaded raw RP data file through a post-processing step. The raw RP data file contained a record for each segment number, timestamp, V_T_, intensity, and status flag of the return signal. For each of the 16 segments, two possible status flags could be received, those being “Flag = 1” and “Flag = 35.” Depending on the status flag received for the measurement cycle, the program captured between 20 to 60 observations for the 15-minute time interval. The post processing step creates a single record in the second data file for each 15-minute collection interval using the raw RP data as the input file. The record contains the timestamp; and for each segment the record is appended to include segment number, the mean distance to target and intensity for each flag, the count of the number of measurements for each flag, the total number of measurements, and a percentage of the number of “Flag = 1” returns for the given 15-minute measurement interval (see Figure 3 *Final.csv Data File at the bottom left of the diagram).

The Leddar’s on-board processing chip computes status “Flag = 1” as a valid return signal of a single object detected in the emitted beam. “Flag = 35” is a valid return but is interpreted by the sensor as more than one object detected within the same segment as a result of the enabled “object demerging” configuration setting. “Flag = 35” is considered noise in the data due to an implicit assumption that no objects should exist between the statically mounted Leddar sensor and the ground or snowpack surface. These measurements are retained in the final post processed data file, but they are not used for the snowpack depth calculation. The presence of “Flag = 35” does not mean all data for a given segment are invalid. If the timestamp contains data with the “Flag = 1” status, a valid return signal has been received for the specific segment. As part of the quality control process, the “noisy” (Flag = 35) V_T_ and intensity values are discarded from the specific segment. The count of noisy returns is retained and used in the analysis. 

#### 2.2.2. SR50A Sonic Ranging Device

The SR50A was configured using a Campbell Scientific CR1000 data logger [29] with standardized temperature-adjusted programming for a 15-minute data collection interval for the distance to target measurement. Real-time temperature compensation is performed using the temperature sensor located adjacent to the Leddar and SR50A on the instrument tower (Figure 2).

#### 2.2.3. Meteorological Sensors

Quality assurance corrections were applied to the complete meteorological dataset. The totalizing precipitation weighing gauge accumulates rain and snow in a catchment bucket. It is susceptible to diurnal and long-term drift, evaporation, the under-catch of snow caused by wind during a precipitation event, and the over-catch from blowing snow [34,35]. Totalizing precipitation weighing gauge data adjustments followed the methodology described by [36,37]. Initial manual cleaning was completed to correct gauge measurements resulting from sensor maintenance tasks such as emptying liquid when the catchment bucket was full. To correct for diurnal, long term drift, and evaporation, negative and small positive changes were removed using a threshold of 0.11 mm unless a precipitation event was in progress in prior and subsequent measurements. To reduce overestimation of under catch above the sensor, the correction of the wind field was limited to speeds greater than 1.2 ms^−1^ and less than 6.5 ms^−1^. Precipitation measurements were calculated using the change in the catchment bucket volume from the previous measurement.

Incoming shortwave radiation (SWI) and reflected shortwave radiation (SWR) were collected from a Campbell Scientific CNR1 [38] net radiometer mounted on the same tower as the Leddar and SR50A units. CNR1 SWI and SWR data were used to calculate albedo (ratio of SWR over SWI) for both a one-hour and daily averaged interval. Measurements greater than one were discarded as SWI must always be greater than SWR. Undulations in the snow surface or other highly reflective objects in close proximity to the CNR1 can produce additional backscatter that is recorded by the sensor [39]. There were five days when the albedo calculation was greater than one. For those days, daily albedo was computed using the mean from the previous and subsequent day. 

### 2.3. Aggregated Datasets for Analysis

The final post-processed 15-minute dataset contained 12,844 records. Snow depth (D_snow_) was calculated for each of the Leddar segments and SR50A sensors by subtracting the baseline “no-snow” (V_o_ = 2.93 m) ground measurements from subsequent “snow” surface (V_T_) measurements (Equation (2)). The mean Leddar D_snow_, V_T_, and intensity were calculated across the 16 segments, where V_T_ is estimated from Equation (1). Several of the Leddar segments possessed “noisy” (Flag = 35) data records, which were removed prior to calculating the means.

D_snow_ = V_o_ − V_T_ (2)

Aggregated datasets were created using quality-controlled data for all sensors to reduce the dataset to a manageable size. A daily timestep was created using the 24-hour mean for all variables. To maintain a higher temporal resolution while retaining diurnal variability, a second data file contained records for the mean of the 15-minute data at a one-hour timestep. The dataset contained 3159 one-hour observations. The “proportion of clean returns” is the total number of one-hour observations containing “Flag = 1” (clean) data relative to the “total number of observations” for the timestep. The calculated percentage is the “total observations” divided by the total number of “clean” observations. The “proportion of noisy returns” is the total number of one-hour observations divided by the total number of “Flag = 35” (noise) one-hour observations. 

### 2.4. Controls on the Leddar Intensity Signal

To investigate the drivers of the Leddar intensity signal through time, a Pearson’s correlation matrix was computed for the hourly dataset to examine relationships and potential collinearity between variables. Variables selected were wind speed (WS), daily albedo, air temperature, relative humidity (RH), ground temperature, SR50A snow depth, hourly precipitation, the Leddar V_T_, intensity, and proportion of clean returns per timestep. Univariate statistical analysis was completed for the Leddar intensity and proportion of clean return signals for air temperature, daily albedo, V_T_, and relative humidity after being identified as “variables of interest” in the correlation matrix. The correlation analysis was limited to only sample periods when complete snow cover was present beneath the Leddar unit (21 December 2017 to 27 April 2018). 

### 2.5. In-Situ Evaluation of LeddarTech IS16 Sensor’s Precision, Accuracy, and Performance

An initial test of the Leddar sensor precision was completed in the field on 14 December 2017 before the start of the 2017–2018 data collection. A 1.22 m × 2.44 m painted plywood reflective target used by [40] for LIDAR radiometric calibration was placed level on the ground to compensate for the sloped surface under the Leddar sensor to increase the intensity of the return signal. Leddar DTT and intensity data as well as SR50A DTT measurements were captured in 15-minute increments for a four-hour time period. The SR50A and the Leddar segment 16 beams did not fall fully within the target surface due to the sensor field of view being larger than the target dimension. The Leddar beam length was 2.61 m, which exceeded the painted target’s length of 2.44 m. Leddar status “Flag = 1” observations were used to calculate the mean distance to target and intensity for each of the 16 segments. 

A second analysis was completed to evaluate the precision of the Leddar unit when operating over a snowpack surface under completely stable conditions; i.e., during a period when the snowpack was in a stable state such that settling, compaction, and crystallization had no significant impact on sensor observations. Several criteria were used to select a time period to test for consistent measurement in snow depth once snowpack settling had taken place. Influences from solar radiation were removed by selecting a sampling interval between 20:00 to 06:00. The air temperature was less than –5.0 °C prior to and throughout the sampling interval to avoid the melting and refreezing metamorphoses of crystalline structures [14,16]. No precipitation event occurred for several days prior to observation to remove accumulation, compaction, and settling influences [21]. Wind speeds approaching zero were desired to remove redistribution of the snowpack surface [15]. Due to an unusually warm winter with few days between precipitation events, there was only one time period from 26 December 2017 to 27 December 2017 meeting these criteria. 

## 3. Results and Discussion

**Figure 4:**
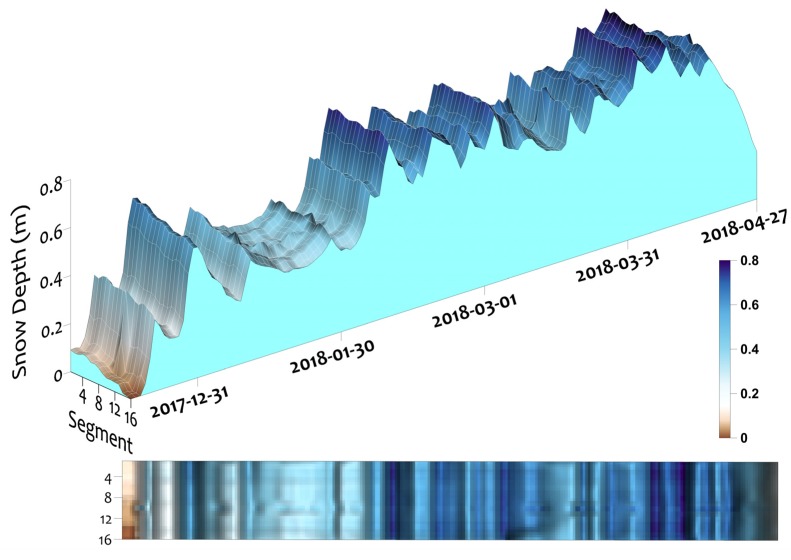
Leddar snow depth 15 December 2017 to 27 April 2018.

The Leddar daily data were used to plot both snowpack depth and intensity for the winter 2017–2018 snow season. Figure 4 shows snowpack depth variability across the 16 segments of the sensor footprint, while increases in depth through time represent the snow fall accumulation events. Settling, compaction, or a mid winter melt are seen as decreases in depth over time. Snowpack surface morphology and texture within the sensor field of view are illustrated orthogonal to the time axis. The rapid reduction in snow depth at the end of the time series was due to spring melting as air temperatures and day length increased in mid to late April. 

**Figure 5:**
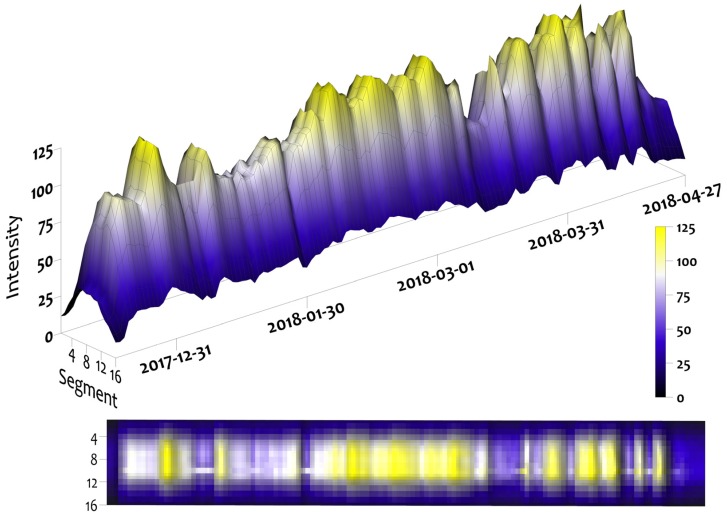
Leddar intensity return signal from 15 December 2017 to 27 April 2018. Before the implementation of all of the quality control steps.

The Leddar intensity for the 16 segments is shown in Figure 5. At the beginning and end of the series, low intensities are associated with patchy snow combined with bare soil and vegetation at the ground surface level. Higher intensity values occur, when snow cover completely fills the sensor field of view. During the period of 100% snow covered area (SCA), intensity increases immediately following snow accumulation events and then gradually decreases as the snowpack settles and metamorphoses. These patterns of increasing and decreasing intensity are synchronous with increases and decreases in depth, but the magnitudes of depth and intensity change are not visually correlated. Intensity responses are strongest at nadir segments with decreasing values toward the outer edge of the field of view. The laser radar equation shows there is an inverse relationship with DDT and the intensity response [32,41]. Segments at nadir are closest to the ground surface and have the highest intensity. For each segment starting at nadir going to the edge segments of the sensor, distance to the ground increases and there is a corresponding decrease in intensity.

### 3.1. LeddarTech IS16 Sensor Performance

#### 3.1.1. Signal Data Noise

The Leddar DTT measurement is dependent on the “clean” (“Status Flag = 1”) return signals and intensity amplitude. Due to the Leddar demerging configuration setting being “enabled,” the sensor occasionally recorded signals with a “Flag = 35” status (noisy returns). Analysis was completed for the entire hourly time series (14 December 2017 to 27 April 2018) dataset to evaluate the amounts of “clean” (proportion of clean returns) and “noisy” (proportion of noisy returns) return signals for all segments (Table 3a). The proportion of timestep observations with no clean returns (Table 3b) is the amount of missing clean data for the individual 15-minute data sampling measurements. Intensity (Table 3c) for segments near nadir received the highest values, while edge segments received the lowest intensity measurements. Of note, the two edge segments on both sides of nadir had minimum intensity values below the manufacturer’s specifications for DTT accuracy threshold which occurred during the time period of no-snow. Segment 10 had an abnormally high noise fraction throughout most of the observation period. This segment does not appear to be representative of the sensor. The noise was potentially caused by internal damage or contamination within the optical receiver.

**Figure 6:**
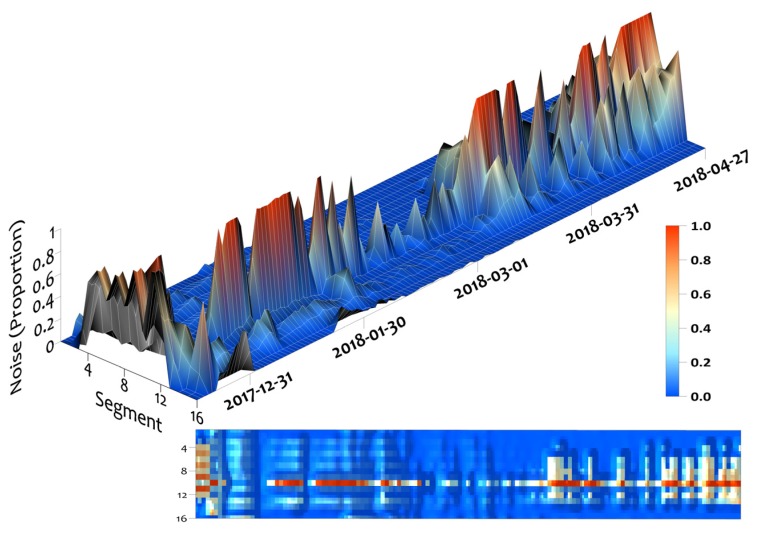
Leddar “noise” in the data from 14 December 2017 to 27 April 2018. The noise is the proportion of the data per time step that received noisy returns in relation to the total number of returns. Time series of readings culminated in Table 3a—proportion of noisy returns.

The noise was plotted over time to find potential relationships with snowpack conditions and meteorological influences. Figure 6 shows the proportion of the data per time step that received noisy returns in relation to the total number of returns. The least amount of noise was observed in the middle of the series once the SCA was fully present at the end of December and before melt conditions initially occurred in mid-March. Segments near nadir experienced more noise during episodic snowmelt periods late in the winter season, when there was higher water content in the snowpack. Some noise may have been caused by solar contamination, precipitation, wind redistribution of snowpack surface grains, or higher moisture content when the pack entered a freeze/thaw stage or became isothermal and melted out. 

#### 3.1.2. Temperature Sensitivity 

**Figure 7:**
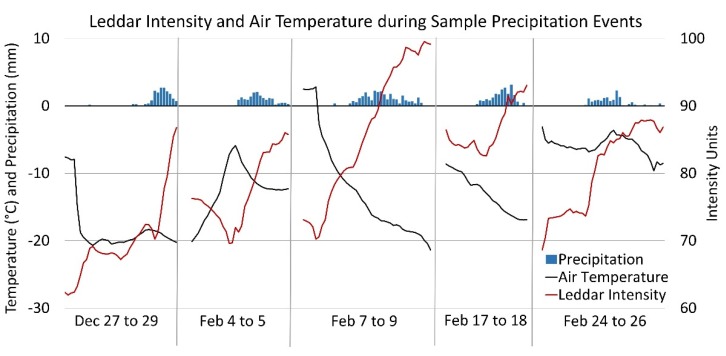
Leddar intensity (mean all segments), air temperature, sample precipitation events. Initial examination of the Leddar intensity signal suggests an LED light output sensitivity to air temperature.

The high-resolution time series data demonstrated the intensity amplitude shifted by 10 or more units immediately before some snowfall events (Figure 7). This indicated at least some of the shift in signal intensity associated with snowpack accumulation events was not a function of the snowpack surface condition but instead was impacted by the inverse relationship between LED light output power and ambient temperature [42]. Figure 7 show five events (precipitation: blue bars; air temperature: black; intensity: red) with increases and decreases in the intensity response prior to and during the events. These data show that signal intensity tends to vary inversely with air temperature leading into the precipitation event, and is likely a hardware response demonstrating some thermal sensitivity within the Leddar unit. Consultation with the manufacturer confirmed both internal hardware components and environmental conditions influence the sensitivity of the Leddar intensity signal [28]. Further analysis is required to separate out the ambient temperature influence on the hardware vs. the surface reflectance response; however, this preliminary illustration of an inverse relationship with temperature suggests that temperature-based correction of the intensity response, while not implemented here, could be achievable. 

#### 3.1.3. Controls on the Leddar Intensity Signal

The full winter season 1-hour dataset with Leddar intensity signal, proportion of clean returns, air temperature, daily albedo, and Leddar V_T_ data show moderate to strong correlations (Table 4). 

The daily albedo and Leddar intensity return signal show a correlation of 0.77. There should be a relationship between these two variables as they are both a measure of reflectance. Higher reflectance values are received from fresh dry snow when smaller snow grain sizes are present [43]. Lower reflectance values occur as the snowpack metamorphoses as a result of changes in crystal structure and increased snow grain size [22,44]. Reflectance of aged, wet, and melting snow is lower than that of fresh, dry snow [21,45]. Ice layers formed from melt/freeze cycles have similar reflectance properties to that of wet snow [12,19]. Albedo and the proportion of clean Leddar returns showed a moderate correlation of 0.59, which indicates that noise tends to diminish as snowpack reflectance increases. 

There is a correlation of −0.77 between the air temperature and the Leddar intensity signal (Table 4). In addition to potential hardware influences (discussed above), air temperature also influences the snowpack properties of grain size and structure [16]. As air temperatures increase and approach 0 °C, the snowpack begins to ripen (higher water content) and surface reflectance reduces [46]. 

There is negative correlation between air temperature and the proportion of clean Leddar returns at −0.57 (Table 4). The proportion of clean returns was elevated during cooler temperatures. As air temperature approached 0 °C and warmer, more noise was present in the data. This could be influenced by another driver that auto-correlates with seasonal variations in temperature, such as freeze/thaw conditions at the surface of the pack during the melt phase, suggesting noise levels increase as the snow surface melts. 

No relationship exists for V_T_ and the clean return signal (*r* = −0.13), but V_T_ and intensity demonstrate a correlation of −0.43. The further the target is from the sensor, the less backscatter received [47]. Since the Leddar unit was statically mounted on a tower facing the ground surface, the largest DDT values occur before the snowpack is present. At this point, the sensor is observing the vegetation and soils which have a lower spectral reflectance than snow in the NIR 940 nm band. As the snowpack builds, DDT is reduced, and Leddar intensity increases due to the higher albedo of snow, as well as the shorter range. As the pack starts to melt out, spectral reflectance values decrease from the higher water content in the snowpack as well as the increased distance to the ground surface.

**Figure 8:**
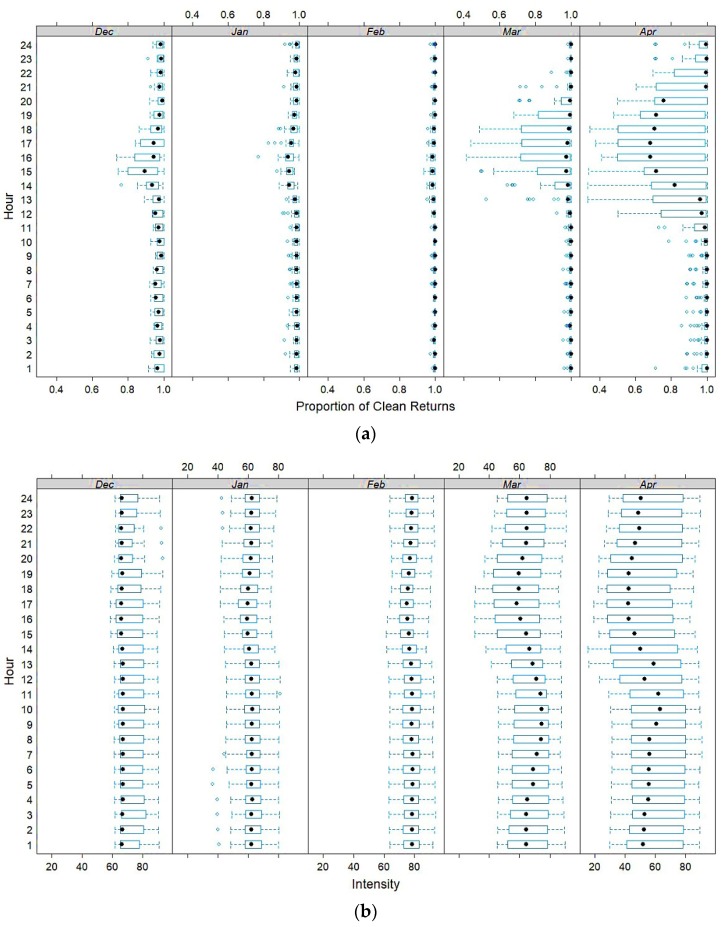
Proportion of clean signals (**a**) and mean hourly Leddar signal intensity (**b**) by month from December to April. The box delineates the lower 25^th^ and upper 75^th^ percentiles. The dashed T-lines are the minimum and maximum values and open circles to the left and right of the dashed T-line are outliers. The black dot inside the box is the mean.

Intensity and proportion of clean returns are positively correlated (*r* = 0.74, Table 4). The intensity signal and proportion of clean returns were plotted through time once the snowpack emerged. Box plots in Figure 8 are broken down by hour-of-the-day and further by month. Both the proportion of clean returns (Figure 8a) and the intensity signal (Figure 8b) show diurnal patterns. Signal “noise” and intensity ranges are elevated during daylight hours from midday onwards, which appear to correspond with temperature and daylight variations. Maximum noise and lowest intensity are associated with warmer afternoon temperatures, while minimal noise and slightly elevated Leddar reflectance occur in the morning after sunrise but during local diffuse sky radiation. The pattern was more dominant in March and April, when most daytime air temperatures were above 0 °C. The least amount of noise occurred in February when air temperatures were between −20 to −35 °C with only a few days approaching or above 0 °C. There are two potential contributing factors: (i) occasional melt, ripening processes, and higher water content at the surface of the snowpack; and/or (ii) solar contamination of the signal during the afternoon. With the diurnal variation in sensible heat flux and net surface radiation balance over the snowpack, it is possible the Leddar signal is sensitive to the changing snowpack surface structure and increasing snow grain size. There is a corresponding increase in noise levels in March and April when diurnal energy inputs to the pack can be most extreme. Any sensitivity to changes in surface structure, would be expected to be observed during the onset of melt conditions late in the season. 

Solar contamination cannot be ruled out, however, as the skyview surrounding the instrument tower is most open to the west with mountain ridges dominating the south and north skyline. Consequently, if solar contamination occurs, it would be expected in the afternoon, with the level of contamination increasing as the solar zenith and range in azimuth increase later in the season. It is believed that both surface freeze/thaw processes and solar contamination play a role in influencing diurnal intensity and noise patterns, but further investigation is required to quantify the relative influence and identify which is dominant. 

### 3.2. Range and Depth Observations

#### 3.2.1. Leddar Calibration

The root-mean-square-error (RMSE) of range-to-V_T_ calibration conducted in the laboratory using Equation (1) was 13.0 mm. The parameter values (and standard error): β, 3.134° (0.010°); γ was −0.044° (0.003°); and R_c_ was −0.307 m (< 0.001 m). The calibrated β was just over the LeddarTech product specification of 3.0° [24].

**Figure 9:**
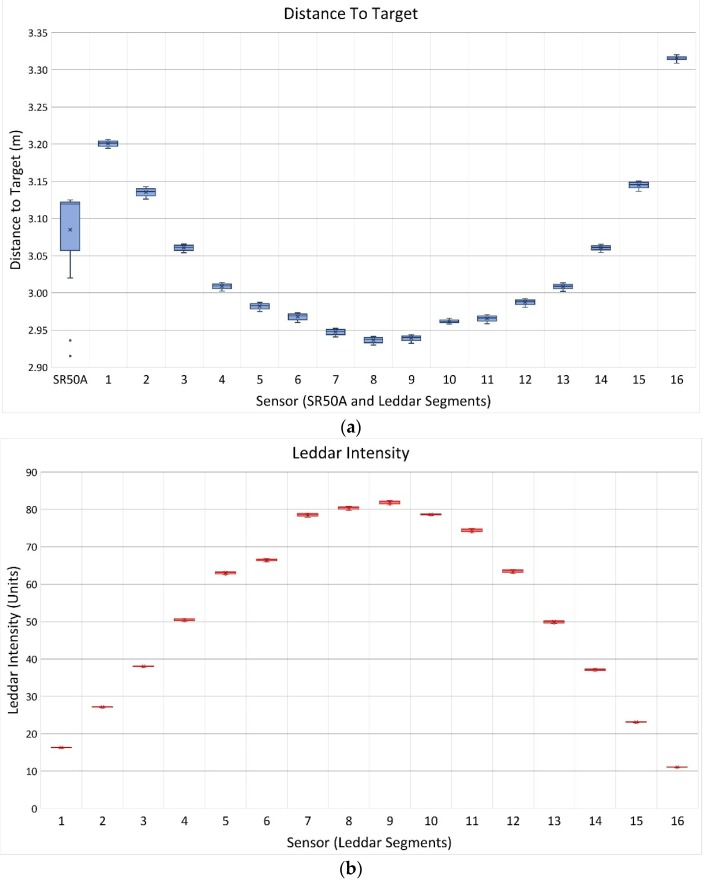
Reflective target placed on the ground under the Leddar unit. SR50A footprint and Leddar Segment 16 were not contained within the target surface. (**a**) Boxplot of Leddar and SR50A DTT, (**b**) boxplot of Leddar Intensity. (Note: SR50A does not return intensity signal measurements).

Results of the Leddar and SR50A reflective target testing from 14 December 2017 (as described in Section 2.5), are shown in Figure 9. The Leddar DTT follows a systematic arc from the optical receiver to the target surface [24]. Segment DTT is shortest at nadir and increases toward the edge segments. The range of DTT values show consistent measurements for all segments within 20 mm and a maximum standard deviation for all segments of 5 mm (Figure 9a) over a four-hour duration. The SR50A measurement varied to a higher degree with a DTT range of 210 mm and a standard deviation of 70 mm. Under controlled conditions, variation in the Leddar measurements was very low and more precise than the SR50A. The intensity signal (Figure 9b) follows an expected systematic inverse pattern with highest values at nadir and decreasing towards the outer segments. The footprint for segment 16 was at the edge of the target and was contaminated with bare soil and grass returns, resulting in the lowest intensity relative to other segments. The intensity measurement over the four-hour period for segment 16 was 11.1 which was below the Leddar manufacturer’s threshold of 15. However, the DTT standard deviation for segment 16 was 3 mm. The temporal stability of measurements from this Leddar sensor testing are consistent with the findings of [48] in their preliminary investigation of the Leddar technology. 

#### 3.2.2. Snow Depth Validation

The full winter season 1-hour Leddar and SR50A depth dataset contained 3211 records. Table 5 shows snowpack depth for the SR50A and each Leddar segment when the snowpack reached maximum depth on 8 April 2018 at 10:00. Snow depth varies across the Leddar field of view with lowest measurements of 0.75 m in segments 5 through 7 and largest observations of 0.85 m for segments 14–16. This difference is easily within the expected variance associated with the slightly sloping ground surface and undulations in the snowpack surface. The snow depth recorded for the SR50A was 0.83 m. The SR50A measurement falls within the range of the Leddar snow depth for segments 13 and 14. SR50A observations are not necessary expected to be the same as any individual Leddar segment due to the size and shape of the different sampling footprints (Figure 1). 

Comparable Leddar, SR50A, and field validation data are presented in Table 6. The Leddar “Proportion clean returns” reduces as air temperatures increase during melt conditions. The Leddar footprint shows a range in snow depth from a low of 0.08 m to a high of 0.17 m across all segments. The SR50A snow depth measurement was between zero to 0.04 m for all site visits except the last when significant melt occurred in a short period of time.

**Figure 10:**
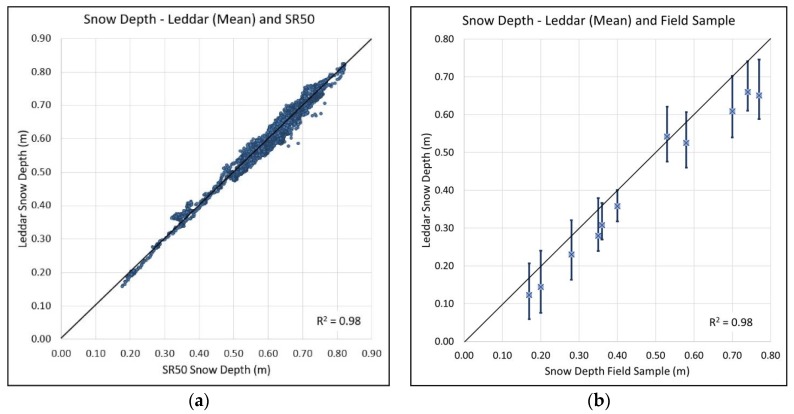
(**a**) Plot of Leddar segment mean against SR50A mean snowpack depth. (**b**) Plot of Leddar segment depth range (bars) and mean (blue square) against manual field measurements.

A regression plot of Leddar and SR50A snow depth (Figure 10a) shows strong agreement between the two sensors (*r*^2^ = 0.98). Figure 10b shows the mean for the Leddar snowpack depth values across all segments plotted against the single field snow depth measurement below the Leddar unit. There is low confidence that the manually collected field data represent the whole Leddar or SR50A sampling footprint. Single manual sampling depth observations were obtained from the back of the tower outside the sensor footprints reaching approximately 1 m to the location under the Leddar unit attempting to minimize snow surface disturbance. The manual measurements fall within the Leddar segment array distribution range as seen in Table 6 but were not co-located to any individual segment. The Leddar segment means tend to fall below the field measurement resulting in a bias on the order of ~0.05 m due to variability in snow depth among all segments or manual field measurement errors such as the probe not penetrating to the ground surface from ice layers at the base of the pack. However, despite this small bias Leddar and field depth data show a strong regression (*r*^2^ = 0.98).

#### 3.2.3. Leddar Snow Depth Stability 

The night-time sampling interval that met the stable sampling constraints of air temperatures consistently below −5 °C with no precipitation or wind, was a 10-hour period between 20:00 on 26 December 2017 to 06:00 27 December 2017. Table 7 shows snow depth for the Leddar segments and SR50A. The range in snow depth measurement of each individual Leddar segments was <5 mm, while the range in SR50A snow depth over the single footprint was >20 mm. The precision observed for the Leddar sensor exceeds the manufacturer quoted 6 mm when intensity is greater than 15 [24]. 

## 4. Conclusions

This proof of concept study examined the feasibility of using a LeddarTech IS16 LED LIDAR-based sensor for snowpack surface morphology, depth, and reflectance measurements. The sensor was co-located and tested against a Campbell Scientific SR50A sonic ranging snow depth sensor during the 2017–2018 winter season. Controls on the intensity signal, comparison to SR50A snow depths, Leddar distance-to-target (DTT) precision, and accuracy were evaluated. 

The Leddar sensor data showed variability in magnitude of the intensity signal during the formation, settling, ripening, and ablation of the snowpack as well as when precipitation events took place. Prior to snowpack formation, vegetation and soils produced more noise in the dataset as well as lower intensity responses. Lower intensity and increased noise also occurred during surface melt conditions. More noise was present in segments closer to nadir than at the edges of the Leddar footprint. Diurnal variations in the intensity and noise levels increased later in the season, suggesting that solar contamination of the Leddar signal could be present and that mitigation measures need to be explored. There was a high correlation between albedo and the intensity signal (0.77) as well as noise (0.59). Air temperature demonstrated a negative correlation (−0.77) with intensity, which was likely at least partly a function of hardware sensitivity, though it could also be influenced by surface melt lowering the intensity response. Further analysis is required to quantify and better understand the impact of air temperature on the intensity signal. 

The Leddar sensor data showed variability in snow depth measurements across the 16-segment footprint during snowpack accumulation, compaction, and ablation events. Examining sensor performance when the snowpack was in a stable state showed Leddar snow depth observations varied <5 mm in comparison to >20 mm for the SR50A measurements. These Leddar observations exceed the manufacturer’s specification for accuracy at 50 mm and precision of 6 mm for intensity values greater than 15. Despite the environmental sensitivities experienced by the Leddar unit, the sensor demonstrated accurate and precise depth measurements throughout the winter season. The Leddar and SR50A snow depth measurements displayed a strong linear relationship (*r*^2^ = 0.98), with virtually no bias and a slope of 0.99. Leddar and manual field measurements also showed strong agreement (*r*^2^ = 0.98). Leddar measurements during initial testing using a reflective target were within 20 mm for DTT, compared to 210 mm for the SR50A sensor. 

LED LIDAR has great potential for low-cost monitoring of a range of snowpack conditions, including depth measurements for 16 independent beam segments, surface morphology, and possibly surface reflectance if temperature influences on the internal optics can be mitigated. The base components used in this study were obtained for a combined approximate cost of <CA$1000, which is similar to a new sonic ranging sensor. Meanwhile, *in-situ* Leddar snow depth observations can be made at higher precision and accuracy, over multiple segments along a transect, while the intensity signal and noise response show promise for either characterising snow surface properties and/or perhaps precipitation. Compared to traditional laser-based ranging sensors, LED LIDAR has a low intermittent operational power requirement, making it a suitable candidate for remote deployment where power options are limited. 

## Figures and Tables

**Table 1:** Leddar and SR50A sonic ranger manufacturer technical specifications [24,29].

Sensor	Leddar IS16	SR50A Sonic Ranging Device
Type	Leddar LED Multichannel LIDAR sensor, built-in processing chip performing proprietary temperature adjusted Full-Waveform analysis for multi-object detection distance measurement	SR50AA Sonic Ranging Sensor with independent temperature compensation
Manufacturer	LeddarTech Inc.	Campbell Scientific (Canada) Corp.
Distance	0 to 50 m	0.5 to 10 m
Operating Temp	−40 °C to +50 °C	−45 °C to +50 °C
Accuracy	±50 mm Quoted for a moving target	±10 mm or 0.4% of DDT (greater value)
Precision	6 mm (manufacturer specification if intensity > 15)	
Resolution	10 mm	0.25 mm
Measurement Rate	Up to 50 Hz	Less than 1.0 second
Emitter	Single LED diffused light source beam	Sonic Ranging ultrasonic pulses
Receiver	Measurement of backscatter on a 16-Channel photodetector array	Listening for return echoes
Beam Length	48° (Distance from sensor * 0.8905)	30° (Radius = 0.268 * Height)
Segment Length	1/16 of the Beam Length (Beam Length / 16)	N/A
Beam Depth	8° (Distance from sensor * 0.1402)	N/A
Wavelength	940 nm (infrared)	50 kHz (Ultrasonic) electrostatic transducer

**Table 2:** Leddar measurement configuration settings used for the 2017–2018 snow season.

Parameter	Configuration	Description
Distance Units	cm	Unit of measurement for distance to target
Accumulations	1024	Range: 0 to 1024. Higher values enhance the range for DTT below 10 m, reduce the measurement rate and noise
Measurement Rate	1.5625 Hz	Range: 1.5625 to 50 Hz. Rate of signal measurement. Lower values give highest accuracy and precision (also known as Refresh Rate)
Oversampling	8	Range: 1–8. High values reduce measurement rate and increase accuracy
Point Count	12	The number of base sample points
Threshold Offset	0.00	Range: −5% to 100%. Modifies intensity threshold. At 100%, no detections. Negative values increase likelihood of false measurements.
LED Control	Automatic	LED power level setting
Change Delay	1 (640 ms)	Number of measurements before sensor changes LED power level
Object Demerging	Enabled	Indicates detection of multiple objects in return signal
Crosstalk Removal	Enabled	Degradation compensation from object detections in other segments
Useful Range	21.3 m	Leddar sensor computed value based on configuration settings

**Table 3:** From 14 December 2017 to 27 April 2018, n = 3211 (1-hour timestep) per segment (SEG) for the entire data collection period. (**a**) Leddar data series proportion of clean and noise returns. (**b**) The proportion of timestep observations with no clean returns refers to the amount of data for all 15-minute measurement cycles where the sensor did not detect any clean readings. (**c**) Leddar intensity signal minimum, maximum, mean, and range.

SEG	Proportion of Clean Returns	Proportion of Noisy Returns	Proportion of Timestep Observations with no Clean Returns	Intensity			
				Min	Max	Mean	Range
1	99.2%	0.8%	0.0%	10.0	45.2	29.3	35.2
2	99.7%	0.3%	0.0%	13.7	61.6	40.8	47.9
3	97.5%	2.5%	0.0%	18.6	82.0	55.0	63.4
4	96.4%	3.6%	0.1%	24.8	105.1	70.9	80.3
5	94.6%	5.4%	0.1%	26.0	123.2	83.4	97.2
6	91.1%	8.9%	2.1%	30.3	128.0	87.8	97.6
7	88.9%	11.1%	2.2%	32.1	134.0	92.6	101.9
8	84.9%	15.1%	5.6%	31.6	131.7	92.7	100.1
9	81.7%	18.3%	5.5%	31.3	134.5	94.8	103.1
10	33.4%	66.6%	38.5%	29.2	135.3	105.1	106.1
11	87.5%	12.5%	1.2%	29.3	136.1	94.3	106.8
12	84.8%	15.2%	5.9%	24.6	118.8	83.9	94.2
13	88.4%	11.6%	0.3%	20.4	96.1	66.3	75.7
14	97.0%	3.0%	0.0%	17.6	75.6	51.9	58.0
15	98.5%	1.5%	0.4%	12.9	57.8	39.7	44.8
16	96.9%	3.1%	1.4%	7.7	38.3	26.0	30.6
(**a**)			(**b**)	(**c**)			

**Table 4:** Pearson’s Correlation coefficient (*r*) for the Leddar (mean of all segments) sensor and other meteorological variables for the given timestep. “% Clean” is the proportion of clean returns.

	Daily Albedo	Air Temperature	Leddar V_T_	Leddar Intensity
	*r*	*r*	*r*	*r*
**Leddar Intensity**	0.77	−0.77	−0.43	-
**Leddar % Clean**	0.59	−0.57	−0.13	0.74

**Table 5:** Maximum snowpack depth occurred on 8 April 2018 10:00. Leddar segments and SR50A snow depth. From 14 December 2017 to 27 April 2018 winter season 1-hour dataset, n = 3211.

SR50A	Leddar Segment (m)															
(m)	1	2	3	4	5	6	7	8	9	10	11	12	13	14	15	16
0.83	0.81	0.79	0.80	0.77	0.75	0.75	0.75	0.76	0.77	0.75	0.78	0.78	0.81	0.85	0.85	0.85

**Table 6:** Manual field measurement, SR50A, Leddar (all 16 segments), mean air temperature, and daily albedo. Manual snow depth measurements taken from behind the tower out of the sensor footprints under the Leddar unit, but exact location varied with each visit. All data collected on calm days with no precipitation.

Site Visit Date	21-Dec	05-Jan	20-Jan	21-Feb	04-Mar	09-Apr	15-Apr	19-Apr	26-Apr	27-Apr	27-Apr
Start Time	18:30	11:00	15:00	10:45	14:45	14:00	14:00	18:15	06:00	07:00	11:15
End Time	22:30	15:00	19:00	14:45	18:45	18:00	18:00	21:45	10:00	11:00	12:30
Snow Depth (m)											
Manual Field Sample	0.35	0.40	0.36	0.70	0.74	0.77	0.53	0.58	0.28	0.20	0.17
SR50A Mean	0.31	0.44	0.36	0.66	0.72	0.71	0.59	0.57	0.29	0.21	0.17
SR50A Min	0.29	0.42	0.35	0.65	0.72	0.70	0.59	0.56	0.28	0.20	0.06
SR50A Max	0.31	0.45	0.36	0.69	0.72	0.73	0.60	0.58	0.29	0.22	0.20
											
Leddar Mean	0.28	0.36	0.31	0.61	0.66	0.65	0.54	0.53	0.23	0.14	0.12
Leddar Min	0.24	0.32	0.27	0.54	0.61	0.59	0.48	0.46	0.16	0.08	0.06
Leddar Max	0.38	0.40	0.37	0.70	0.74	0.75	0.62	0.61	0.32	0.24	0.21
Proportion clean returns	92%	83%	81%	93%	93%	59%	47%	49%	79%	63%	28%
Air Temperature (°C)	−6.2	3.2	−0.3	−13.4	−7.4	7.4	7.5	5.0	4.8	5.1	16.8
Daily Albedo	0.84	0.83	0.80	0.81	0.85	0.77	0.69	0.73	0.59	0.58	0.58

**Table 7:** Leddar and SR50A sensor snow depth data from 20:00 26 December 2017 to 06:00 27 December 2017 nighttime sampling period meeting the minimum specifications.

Leddar	Snow Depth (m)				
Segment	Mean	STDev	Min	Max	Range
1	0.191	0.001	0.190	0.194	0.004
2	0.181	0.001	0.180	0.183	0.003
3	0.189	0.001	0.188	0.190	0.002
4	0.179	0.001	0.178	0.181	0.003
5	0.157	0.001	0.156	0.158	0.002
6	0.162	0.001	0.161	0.164	0.003
7	0.161	0.001	0.160	0.163	0.003
8	0.174	0.001	0.173	0.176	0.003
9	0.179	0.001	0.178	0.182	0.003
10	0.169	0.001	0.168	0.172	0.003
11	0.173	0.001	0.172	0.175	0.003
12	0.176	0.001	0.175	0.178	0.003
13	0.191	0.001	0.189	0.193	0.003
14	0.212	0.001	0.211	0.215	0.004
15	0.197	0.002	0.195	0.200	0.005
16	0.211	0.001	0.210	0.214	0.004
**SR50A (m)**	0.174	0.008	0.164	0.185	0.021
